# Supplementary material for: Differences in Formation of Prepuce and Urethral Groove During Penile Development Between Guinea Pigs and Mice Are Controlled by Differential Expression of Shh, Fgf10 and Fgfr2
Source: Cells. 2025 Feb 27;14(5):348. doi: 10.3390/cells14050348 (PMC11899664; doi:10.3390/cells14050348)
Supplement: Supplementary file 1 [file cells-14-00348-s001.zip › cells-3432277-supplementary.pdf]

**Supplementary Materials:**

**Table S1**. PCR primers used to clone guinea pig genes for making RNA probes.

| Gene | Forward (from 5′ to 3′) | Reverse (from 5′ to 3′) | Accession No. |
|---|---|---|---|
| *Bmp4* | GGAGCTTTCACCACGAAGAA | CCTCTACCACCATCTCCTGATA | XM_023565919 |
| *Bmp7* | CAGCCGAATTCCGGATCTATAA | AAGCCACTTGGTGCTCAA | XM_003463644 |
| *Fgf8* | GACTGTGTGTTCACGGAGAT | GAAGGGTGGGTAGTTGAGAAA | XM_003474949 |
| *Fgfr2* | GATGATGCCACGGAGAAAGA | CCATCCTGTGTCCTTCCTTTAG | XM_063244860 |
| *Hoxd13* | TAACCCTCACCTGGCTCTAA | CTCACAGACATTGCCTCTACAA | XM_023561145 |
| *Ptch1* | GAACCCTCGATCGCATCC | TCCACGTCTTGCAGCTCTAT | XM_063227398 |
| *Wnt5a* | GCCCAGGACCCACTTATTTAT | CAACACATCCCTCCCAAAGA | XM_013143894 |

**Table S2**. QPCR primers for genes in developing genital tubercles of mice and guinea pigs.

| | Gene | Forward (from 5′ to 3′) | Reverse (from 5′ to 3′) | Accession No. |
|---|---|---|---|---|
| Guinea pig | *Shh* | CCAGAAACTCCGAGCGATTTA | CCAGGGCATTTAATTTGTCCTTAC | XM_003469458 |
| | *Bmp4* | TCTGTCAATTCCAGCATCCC | CCTCTACCACCATCTCCTGATA | XM_023565919 |
| | *Fgf8* | GCTGAGACTGGTCTCTACATTT | TTGTTCTCCAGCACGATCTC | XM_003474949 |
| | *Fgf10* | TTGCCTCTGTGGGAAGTATAAG | GTAGGAGGAGGGAGTGATTCT | XM_003470193 |
| | *Fgfr2* | GTGCTTGGCGGGTAATTCTA | TCTTGGTCGTGGTCTTCATTC | XM_063244866 |
| | *Hoxa13* | CTGGAACGGCCAAATGTACT | TCTCCGTTTGTCCTTGGTAATG | XM_005008377 |
| | *Hoxd13* | CACTTCGGCAACGGCTATTA | CACGTCCATGTACTTCTCCAC | XM_023561145 |
| | *Ctnnb1* | CCGTTGTGAACCTGATCAACTA | GACCATCACTGCAGCCTTATTA | XM_063260961 |
| | *Actb* | TCCCTGGAGAAGAGCTATGA | CAGGATTCCATACCCAGGAAG | NM_001172909 |
| | *Gapdh* | ACAGTGACAGCCATTCTTCC | AGCCGAACTCATTGTCATACC | XM_063237559 |
| Mouse | *Shh* | GGATGAGGAAAACACGGGAGCA | TCATCCCAGCCCTCGGTCACT | NM_009170 |
| | *Bmp4* | GCCGAGCCAACACTGTGAGGA | GATGCTGCTGAGGTTGAAGAGG | NM_007554 |
| | *Fgf8* | TTGGAAGCAGAGTCCGAGTTCG | GCCGTGTAGTTGTTCTCCAGCA | NM_010205 |
| | *Fgf10* | ATCACCTCCAAGGAGATGTCCG | CGGCAACAACTCCGATTTCCAC | NM_008002 |
| | *Fgfr2* | GTCTCCGAGTATGAGTTGCCAG | CCACTGCTTCAGCCATGACTAC | NM_010207 |
| | *Hoxa13* | CCCAAAGAGCAGACGCAGCCT | GTGTAAGGCACGCGCTTCTTTC | NM_008264 |
| | *Hoxd13* | ATCAGCCACAGGGGTCCCATTT | GAGCTGCAGTTTGGTGTAAGGC | NM_008275 |
| | *Ctnnb1* | GTTCGCCTTCATTATGGACTGCC | ATAGCACCCTGTTCCCGCAAAG | NM_007614 |

| | | | | |
|---|---|---|---|---|
| | *Actb* | CATTGCTGACAGGATGCAGAAGG | TGCTGGAAGGTGGACAGTGAGG | NM_007393 |
| | *Gapdh* | CCATCACCATCTTCCAGGAGCG | AGAGATGATGACCCTTTTGGC | NM_001411843 |

**Table S3**. Ct values of selected genes in developing genital tubercles of mice and guinea pigs.

| | **E12.5 mouse** | **E23 guinea pig** | **P-value** | **E13.5 mouse** | **E26.5 guinea pig** | **P-value** |
|---|---|---|---|---|---|---|
| ***Shh*** | 26.25 (0.32) | 28.07 (0.55) | **0.007** | 27.32 (0.29) | 29.28 (0.58) | **0.022** |
| ***Fgf8*** | 28.72 (0.44) | 30.24 (0.71) | **0.031** | 29.66 (0.47) | 31.54 (0.82) | **0.016** |
| ***Fgf10*** | 25.41 (0.39) | 28.63 (0.65) | **0.002** | 26.18 (0.57) | 28.77 (0.63) | **0.007** |
| ***Fgfr2*** | 26.88 (0.35) | 28.92 (0.51) | **0.001** | 25.27 (0.42) | 27.45 (0.51) | **0.004** |
| ***Hoxd13*** | 24.18 (0.27) | 26.44 (0.33) | **0.012** | 23.15 (0.38) | 25.79 (0.46) | **0.006** |
| ***Hoxa13*** | 25.57 (0.36) | 25.88 (0.48) | 0.136 | 25.62 (0.46) | 26.41 (0.55) | 0.088 |
| ***Bmp4*** | 24.88 (0.35) | 25.27 (0.24) | 0.077 | 23.83 (0.26) | 24.52 (0.42) | 0.091 |
| ***Ctnnb1*** | 22.46 (0.45) | 23.15 (0.51) | 0.062 | 23.07 (0.32) | 23.68 (0.49) | 0.084 |
| ***Gapdh*** | 20.42 (0.38) | 19.77 (0.45) | 0.057 | 21.24 (0.41) | 20.56 (0.53) | 0.066 |
| ***Actb*** | 22.27 (0.46) | 21.32 (0.53) | **0.034** | 22.63 (0.37) | 21.78 (0.61) | **0.026** |

Data are showed as mean (standard error), sample size, n=5. P values less than 0.05 are shown in bold.
